# Adapting HIV prevention evidence-based interventions in practice settings: an interview study

**DOI:** 10.1186/1748-5908-4-76

**Published:** 2009-11-23

**Authors:** Rosemary C Veniegas, Uyen H Kao, Ricki Rosales

**Affiliations:** 1 UCLA Department of Family Medicine and Center for HIV Identification, Prevention and Treatment Services, Los Angeles, CA, USA; 2 City of Los Angeles AIDS Coordinator's Office, Los Angeles, CA, USA

## Abstract

**Background:**

Evidence-based interventions that are being delivered in real-world settings are adapted to enhance the external validity of these interventions. The purpose of this study was to examine multiple intervention adaptations made during pre-implementation, implementation, maintenance, and evolution phases of human immunodeficiency virus HIV prevention technology transfer. We examined two important categories of adaptations -- modifications to key characteristics, such as activities or delivery methods of interventions and reinvention of the interventions including addition and deletion of core elements.

**Methods:**

Study participants were thirty-four community-based organization staff who were implementing evidence-based interventions in Los Angeles, California. Participants were interviewed twice and interviews were professionally transcribed. Transcriptions were coded by two coders with good inter-rater reliability (kappa coefficient = 0.73). Sixty-two open-ended codes for adaptation activities, which were linked to 229 transcript segments, were categorized as modifications of key characteristics or reinvention.

**Results:**

Participants described activities considered modifications to key characteristics and reinvention of evidence-based interventions during pre-implementation, implementation, and maintenance phases. None of the participants reported accessing technical assistance or guidance when reinventing their interventions. Staff executed many of the recommended steps for sound adaptation of these interventions for new populations and settings.

**Conclusion:**

Staff reported modifying and reinventing interventions when translating HIV prevention programs into practice. Targeted technical assistance for formative evaluation should be focused on the pre-implementation phase during which frequent modifications occur. Continuous or repeated measurements of fidelity are recommended. Increased technical assistance and guidance are needed to ensure that reinventions are evaluated and consistent with the aims of the original interventions. Providing strategic technical assistance and written guidance can facilitate effective HIV prevention technology transfer of evidence-based interventions.

## Background

Balancing implementation fidelity with ensuring the deliverability and relevance of an intervention to a target population is an important task in the technology transfer of evidence-based programs and practices into 'real-world' settings [1-3]. The United States Centers for Disease Control and Prevention (CDC) recommends adapting human immunodeficiency virus HIV prevention evidence-based interventions' key characteristics -- modifying activities or delivery methods that do not conflict with the core elements or behavioural theory for the intervention -- to better suit organizational or participants' needs. Core elements of these interventions, which are the components considered responsible for their effectiveness, are not to be modified or deleted. Changes to core elements are considered reinvention [4]. To date, the CDC has identified 69 evidence-based HIV prevention interventions [5].

Adherence to intervention core elements is strongly recommended to ensure effective reduction of HIV-related risk behaviours [6,7]. However, the CDC has also recognized that total adherence is a idealized goal in many settings where these interventions are being used with new target populations [4,8,9]. Knowing when community-based organizations make changes and what changes they make will inform efforts to provide timely guidance and technical assistance regarding what kinds of modifications challenge fidelity to the core elements and behaviour change theories of the original interventions. Three versions of HIV prevention intervention guidance were released by the CDC to aid agencies that are implementing evidence-based interventions with planning and executing these interventions [4,8,9]. All versions of the guidance describe the core elements and key characteristics of specific evidence-based interventions that were recommended by the CDC. They also describe adaptation steps, resource requirements, recruitment, policies and standards, quality assurance, and monitoring and evaluation for such interventions.

### Modifications to evidence-based interventions in practice

Among the most widely-used interventions disseminated by the CDC are the interventions Mpowerment and Sisters Informing Sisters About Topics on AIDS (SISTA). Mpowerment has been adapted by at least 75 and up to 150 community-based organizations in the U.S. [10]. The nine core elements of Mpowerment are: recruiting and maintaining a core group of 12 to 20 young gay and bisexual men to design and carry out project activities; recruiting volunteers to help deliver services and to make important decisions about the program; using project coordinators to oversee project activities; establishing a dedicated project space where many of the project activities can be held; conducting formal outreach, including educational activities and social events; conducting informal outreach to influence behaviour change; convening peer-led, one-time discussion groups (M-groups); conducting a publicity campaign about the project within the community; and convening a community advisory board. Reported adaptations made to Mpowerment across the U.S. included significant changes to core elements and key characteristics to suit the implementers' or local needs [10,11]. From 28% to 75% of community-based organizations (CBOs) delivered the intervention to different ethnic groups, age groups, and settings than were specified by the developers. CDC guidance allows for modifications to the activities for coordinators, volunteers, core group members, informal and formal outreach, M-groups, publicity campaign, and community advisory board.

The seven core elements of SISTA are: conducting small-group sessions on session objectives to address the challenges and joys of being an African American woman using modelling and role plays to promote skills development and acquisition; using a skilled facilitator to lead small-group sessions; using culture- and gender-appropriate materials promoting pride and self-worth among African American women; teaching women sexual assertion skills for use with partners and in sexual negotiations; teaching women condom-use skills, positive attitudes, and norms toward consistent condom use, and knowledge of how to put condoms on their partners; discussing the cultural- and gender-related issues that serve as barriers when negotiating safer sex; and, emphasizing their partner's involvement in practicing safer sex [8]. Staff from at least 334 agencies in the U.S. have been trained in SISTA [12]. Recently-released guidance on this intervention describes how to carefully adapt SISTA to the cultural- and gender-related needs of Latinas [13]. CDC guidance allows for modification of the duration of the sessions, setting for delivery, and addition of other discussion topics relevant to African American women.

### Recommended adaptation pre-implementation and implementation steps

To aid community-based organizations in adapting and implementing such interventions as Mpowerment and SISTA, the CDC guidance states that agencies that are planning to adapt interventions must first conduct formative evaluation to define the target population, culture, behaviours, and risk factors that put the target population at risk for HIV [8]. Agencies are encouraged to develop an intervention implementation plan, provide ongoing leadership to the intervention from within the agency, solicit staff feedback and suggestions for addressing delivery problems encountered, provide additional training to staff to be able to deliver the intervention, ensure fidelity to core elements, and monitor client responsiveness to the interventions as part of quality assurance. In practice, agencies that are using CDC-recommended interventions vary in the extent to which they conduct these pre-implementation and implementation steps [14,15]. Compiling a clear picture of changes made during intervention pre-implementation through maintenance phases can inform the development of future guidance for agencies and can illuminate reasons for increased or decreased intervention effectiveness in preventing HIV.

### Technical assistance and guidance for adaptation

Agencies that were directly funded by the CDC for HIV prevention between 2004 and 2009 were able to publicly access written guidance for adaptation, and contact up to 18 organizations that were funded to provide guidance on adapting, implementing, and evaluating interventions [16-18]. At the time that this study was conducted, few of the agencies that were directly or indirectly funded in Los Angeles, California were aware of such available resources [19]. The forms of CDC guidance were released after the Los Angeles agencies had been funded and initiated their adapted interventions.

## Methods

### Design

Key informant interviews were conducted with publicly identified staff who were implementing evidence-based HIV prevention interventions. Potential participants were recruited from publicly available lists of staff at HIV/AIDS prevention service organizations that were implementing HIV prevention interventions, and via recruitment letters and e-mails that were sent to publicly available lists for HIV/AIDS service organizations in Los Angeles County. The first two authors, who provide ongoing technical assistance on HIV prevention and evidence-based interventions to organizations in Los Angeles County, compiled a list of potential participants based on responses to the recruitment postings and on their knowledge of interventions being conducted in the region. The UCLA Institutional Review Board provided oversight of all activities and to ensure compliance with the Helsinki Declaration (Study ID G05-03-025-11).

### Setting and Participants

Participant eligibility criteria were: being employed by an organization that provided HIV prevention services in Los Angeles County; being involved in the review, selection, implementation, or evaluation of evidence-based HIV prevention interventions at their organization; and being willing to participate in two recorded in-person interviews and a brief survey. Receipt of CDC funding was not an eligibility criteria for this study. There were 41 individuals who were contacted for participation. Potential participants were informed about the aims of the study to understand the use of evidence-based interventions for HIV prevention and to develop technical assistance resources for such interventions in Los Angeles County, California. Of the 41 who were contacted, 34 agreed to participate, three declined participation, and the remaining four were ineligible. Of the three individuals who declined participation two declined because they did not consider themselves to be the appropriate person for the interviews and one person was not interested in participating. These 34 staff worked at 22 distinct organizations. Twenty-one participants were female, 10 were male, and three were transgender male-to-female. Eighteen participants were Latino/Hispanic, four were African American, four were Caucasian, one was Asian/Pacific Islander, and seven reported having multiple ethnicities. Ten participants were at agencies that were implementing multiple evidence-based interventions. Fourteen participants had ten or more years of HIV prevention experience, nine had five to ten years of experience, four had three to five years of experience, one had one to three years of experience, and six had six to eleven months of experience. Six participants had either changed positions or had left their original organization by the end of the study. Only one participant did not complete the second interview. Participants were asked to identify the evidence-based intervention on which they spent the most time. Nine participants mentioned the Mpowerment intervention, five participants mentioned SISTA, three participants mentioned Popular Opinion Leader, three participants mentioned Street Smart, three participants mentioned VOICES/VOCES, three participants worked on multiple interventions equally, two mentioned Healthy Relationships, two mentioned Safety Counts, and four participants mentioned other evidence-based interventions [17].

### Data collection

The first and second authors conducted all interviews. The first phase of interviews occurred between December 2005 and May 2006, and the second phase of interviews occurred between August and October 2006. A semi-structured interview drawn from research on the adoption of evidence-based HIV prevention programs was administered to all participants [20,21].

### Analyses

Interviews were transcribed and entered into Atlas.ti version 5 [22]. Iterative coding of interviews alternated between assignment of predetermined close-ended codes and more descriptive open-ended codes. This iterative method enabled the raters to code general implementation steps being taken by the participants (*e.g*., acquiring information, seeking technical assistance) as well to capture more specific activities and delivery methods that were changed (*e.g*., changing the number, duration, or content of sessions). Ten close-ended activities codes corresponding with intervention pre-implementation, implementation and maintenance, and evolution phases were assigned by the first and second authors to transcript segments. These close-ended codes were based on technology transfer categories of activities defined by CDC scientists [6,20]. The pre-implementation codes were: identify need for new intervention, acquire information, assess fit, prepare organization and staff, and secure technical assistance for intervention selection. The implementation codes were: secure technical assistance for implementation, and conduct process evaluation. The maintenance and evolution codes were: support staff for continued implementation, support organization change, and conduct process through outcome evaluation. Inter-rater reliability for the ten close-ended codes was established among three coders with a random sample of three interviews from each interview wave. Kappa coefficients ranged from 0.82 to 1.00, well above the recommended 0.70 level for similar research [23].

Additional open-ended coding was applied to each of the ten activities by the first and second authors to allow examination of specific adaptations. The open-ended codes used in the current study parallel types of adaptations made to a single evidence-based intervention [24]. For example, the pre-implementation activity 'prepare staff and organization' was also coded with regard to the specific action taken by the staff/organization, *i.e*., 'locating the intervention at a site that is accessible'. A total of 392 open-ended codes were created. Of these open-ended codes, 62 were identified by the first two authors as consistent with adaptations as defined in CDC guidance [8]. Two new close-ended codes were created: modifications to key characteristics, and reinvention. The first two authors used these codes to further classify the 62 open-ended codes. The kappa co-efficient of inter-rater reliability for the two codes was good at 0.73. A total of 229 transcript segments were associated with modifications to key characteristics or reinvention as shown in Table 1. There were 184 segments coded as modifications to key characteristics corresponding with 51 open-ended codes. There were 45 segments coded as reinvention corresponding with 11 open-ended codes. The open-codes assigned to each type of adaptation are listed in Table 2.

**Table 1:** Numbers and percentages of transcript segments (N = 229) coded by phase

	Pre-implementation	Implementation	Maintenance
Modify Key Characteristics	76 (72%)	69 (86%)	39 (89%)

Reinvention	29 (28%)	11 (14%)	5 (11%)

Number of segments in phase	105 (100%)	80 (100%)	44 (100%)

**Table 2:** Codes, definitions and examples for adaptation activities reported by participants

Adaptation (close-ended codes)	Activity (open-ended code)
Modification of key characteristics: Changes to activities and delivery methods that can be adapted to meet the needs of the implementing organization or the target population.	Adapt to make culturally appropriate; adapting language used; having materials in language used by target population or community
	Adaptation of intervention forms; adapting existing materials from other agencies
	Adaptation of scheduling of sessions; number of sessions; duration of intervention
	Adapting intervention to be manageable by staff with existing resources and strengths
	Adding activities to session; changing the curriculum; taking core elements of program to make a new program
	Incentives for participation and retention; providing food at intervention sessions
	Integrating intervention with other client services; integrating with other programs and HIV testing; providing comprehensive services and programs
	Locating interventions at a site that is accessible or at other social venues
	Marketing intervention to better appeal to target population or to a community; tailor outreach
	Piloting program with clients and staff
	Providing childcare for women in intervention
	Recruit from existing groups, other programs, gay community events, agency -led events; recruiting partners; recruit online/the Internet

Reinvention: Changes to the core elements responsible for the effectiveness of the intervention. Core elements cannot be deleted, added to or changed.	Add core element to meet funder requirements, *i.e*., to add sessions
	Have open sessions with non-target group members
	Modifying core element; reinvention
	Other ongoing adaptations; adaptations which reduce intervention effectiveness

## Results

### Changes made during pre-implementation

Staff at agencies modified the activities and delivery methods for their interventions as recommended by CDC. As one staff participant who was implementing Healthy Relationships commented, 'what you could do, really, is take the intervention and reduce for example, change the setting, change the time, and in some ways, change the length of the training, by again, not changing any of the core elements'. CDC guidance for Healthy Relationships lists the number and duration of sessions under key characteristics that can be modified, but does not provide additional information on minimum or maximum duration of sessions. Of the four staff who reported piloting their adaptations, one staff who was implementing Healthy Relationships said they 'conducted about three pilots...A five-day training pilot, a two-day weekend training pilot and a three-day Monday through Wednesday pilot. So, after discussing the different pilots, we came to the conclusion that the best thing was a three-day workshop.' Another staff person who piloted another intervention before selecting Mpowerment commented, 'What we did was we didn't know which one [of two interventions] to start with. We did it, and you know I started to get to know the guys through the men's group, and I asked them, 'What do you think about this?' They're like 'well, it's kind of boring'.' Pilot-testing of intervention components for group-level interventions like Healthy Relationships and Mpowerment is explicitly recommended in the CDC guidance. Staff conducting the VOICES/VOCES intervention modified the videos and language used during the intervention because 'those are the English video [and] a heterosexual relationship...and for the gay community that was like...we don't identify with that' and 'the video it's not a very new video. And it's not really representative of the population here in California, here in Los Angeles County. I think it's an East Coast video.' Such efforts conform to the CDC recommendation to ensure cultural competency in conducting interventions. For a staff person who was implementing multiple interventions, 'Spanish speakers are obviously going to feel more comfortable and it just makes sense if you're going to be conducting this intervention in Spanish, you should have it in Spanish.' Several staff reported incorporating material and monetary incentives to attract clients to the intervention, including a staff person conducting Street Smart, 'We used various other types of incentives, like gift cards, giveaways, we tried raffles -- like we tried raffling off like an iPod shuffle, different things like that.' CDC guidance recommends that incentives such as small prizes be provided for Street Smart participants. One staff who was implementing SISTA commented, 'She [the facilitator] goes to rehab homes, or like teen parenting classes, stuff where girls already go in for a service, so it's easier for her to go ahead and get a captive audience.' This adaptation was consistent with adaptations of the SISTA intervention for youth facilities and younger women [25]. A staff person who was implementing Popular Opinion Leader added a community engagement component by 'making sure the core messages were appropriate for the population. Like doing more of the social change kind of things, like making sure that what we are doing speaks to the history of the community, like really reinforcing the strengths with the booster sessions and inviting people in to talk about areas that the community members identify as challenges.' The impetus for this addition was the identified need to tailor opinion leaders' risk reduction messaging for women who engaged in sex work, *i.e*., reducing the number of sexual partners. Engaging sex workers to reduce the number of partners was considered inappropriate for the clients being served by the intervention. By helping women identify challenges to traditional risk reduction messages, they hoped to strengthen these women's ability to have future conversations. CDC guidance for Popular Opinion Leader does not explicitly discuss community engagement as a part of the intervention, but does recommend that reunion meetings or booster sessions be held with opinion leaders to maintain the intervention.

Examples of reinvention noted by staff in this phase were being required by their funders to increase the number of sessions being delivered in the interventions or to add content to sessions. A staff who was implementing Mpowerment noted, 'You had to create a whole curriculum; we had to create a curriculum aside from the curriculum that was already part of the -- what [the intervention] gave us, we had to create a 16-hour training for the guys.' Five of the other eight staff who were implementing Mpowerment made similar comments about being required to make additions to this curriculum. Another staff who was implementing an intervention for women at sexual risk commented, 'they wanted us to add healthy body image, disclosure. In the [intervention] it didn't focus on any of that. So, originally we had to implement that into the curriculum.' This staff person perceived the inclusion of body image and disclosure in the women's intervention as an added core element because the funder required these additions. In other instances, the agencies were required by the funder to deliver the intervention developed for HIV-negative individuals to HIV-positive individuals, as mentioned by a staff who was implementing Popular Opinion Leader, 'the best example for us would be they [the funder] added a prevention for positives component. Ten percent of all our population receiving services have to meet the PHIP [prevention with HIV positives] requirements, but that's not part of [the intervention].' At the time, the HIV prevention strategic plan for Los Angeles County required that organizations deliver interventions to HIV-positive and HIV-negative individuals. No additional funding was provided to reach these groups separately with the interventions. This same staff person reported being required by the funder to add activities for popular opinion leaders that the staff viewed as inconsistent with the intervention, 'When they do their 14 peer conversations, which they're supposed to be 'conversations', they then have to do risk assessments and collect client level identifiers. And that to me is completely not what [the intervention] is about.' CDC guidance for Mpowerment does not explicitly state whether mandatory training components or sessions beyond the single M-group are considered reinvention. The guidance for the women's intervention allows for inclusion of topics relevant to HIV prevention as determined through key informants and focus groups with the target population. Guidance on the Popular Opinion Leader intervention does not reference whether the targeted intervention population can include both HIV-negative and HIV-positive individuals. Notably, none of the staff mentioned consulting with a technical assistance provider or other expert in making these significant changes to the interventions during pre-implementation.

### Changes made during implementation

Reported modifications to the modes of recruitment and retention were consistent with CDC guidance emphasizing strategies that fit with agency clients and organizational practices. Modifications to key characteristics in this phase included the use of incentives other than money to maintain participation as with this staff person implementing Mpowerment, 'We're offering bigger incentives to attend them. Like, for instance, our next big incentive is attend three of our three sessions and you know, we'll take you to Magic Mountain on Gay Night.' This staff person implementing multiple interventions stated 'we have food in every session. And we know that it always helps, especially with the community that we serve. You know, below the poverty level, they need to eat.' CDC guidance recommends the use of food, transportation, and small prizes which are responsive to participants' needs. Staff continued to adjust the duration of sessions during this phase including a staff implementing an intervention for ethnic minority men, 'right now I'm implementing it according to my contract, which is six sessions broken out into two hours a session, one session a day. The last set that I ran, I did two sessions a week, so I ran it three weeks'. Internet use for outreach activities was noted by four staff, including a staff person conducting Popular Opinion Leader, 'the women are starting to be on-line MySpace, TGYouth.net, different web sites on-line. And we're trying to use that type of... that strategy as far as doing like outreach instead of doing it on the street, to do it on-line'. CDC guidance recommends that recruitment be targeted to venues where persons at high risk may congregate and where high-risk behaviours take place. Internet delivery of Popular Opinion Leader is currently being evaluated as part of a CDC-led clinical trial [26]. Another staff person who was implementing Safety Counts commented, 'one of the things that they did was really try to work with captured audiences. One of the strategies that was used to increase the performance of that program was really tie it into other programs, and other services within the agency, and work with drug treatment centers. So, having Safety Counts as an alternative was a way of recruiting people, but also just recruiting more people into the program.' CDC guidance for Safety Counts allows for drug treatment staff to refer clients to Safety Counts if they do not wish to continue receiving treatment. One staff person who was implementing Mpowerment commented on the integration of social activism with the intervention, 'What we have done is recruit the clients through activities that are connected to social activism. An example is in our work with the local neighborhood council, where we're able to pick up some clients by being active in a social cause setting, which is non-HIV related, making the contact, recruitment through the social activism component, to get them into the intake and prevention and testing.' CDC guidance for Mpowerment lists peer-based change and community building among the guiding principles of the intervention.

Reinvention during the Implementation phase was reported by seven staff, and largely reflected changes that were required by their funders. For a staff person who was implementing Mpowerment 'the [intervention groups] they're not one-time-only sessions as far as the [funder] is concerned, it's actually, it has to be three separate sessions, three separate days.' Mpowerment specified only one session for delivery of the M-group core element, but the funder required a three-session M-group. Similarly, a staff person implementing VOICES/VOCES among other interventions was required to add sessions said, 'for example, VOCES, not meant to be a three-session intervention, right? Under the recommendations of the health department, implementing an intervention that was supposed to be one session, and they're delivering it in three sessions, without having any evidence that that's going to make it any more or any less effective.' The CDC Guidance for Voices/VOCES lists delivery of the single session of the intervention as a component which can be modified. However, no example is provided regarding the addition of sessions. Only one staff person described reinvention that was not driven by the funder. This person who was implementing Street Smart said, 'Like session five, a lot of my groups don't respond to it at all and session seven, I've had people stand up and just leave for session seven. So I'm like, maybe I should just not do that session and focus on another session that we could do. Or take out exercises from session seven and put it in session six.' CDC guidance for Street Smart does not indicate whether sessions can be dropped or modified in this manner, but this is considered a reinvention because the facilitator may have deleted intervention content. None of the staff making these changes referenced contacting a technical assistance provider about the reinventions during implementation.

### Changes made during maintenance

Consistent with quality assurance recommendations in the CDC guidance, staff reported ongoing efforts to improve upon activities and delivery methods for their interventions. A staff person who was implementing SISTA noted, 'we need specific authorization to implement it as a probably, four, two sessions back to back, because it may be that this is what is needed. We need that flexibility to implement them.' CDC guidance for SISTA does include suggestions for shortening or lengthening individual sessions, but does recommend that all five sessions be conducted. One staff person who was implementing VOICES/VOCES commented, 'we were able to expand having our sessions not only here, but in different places like clients' houses, whenever they gather some friends, so someone would volunteer their house just to host the sessions, or going to rehab programs and have a session there.' CDC guidance includes the recommendation that interventions be delivered where individuals at high risk may gather. VOICES/VOCES has been successfully delivered in non-clinic settings, including a neighbourhood center and clients' homes [27]. Other staff reported efforts to enhance staff skills or client engagement, such as the staff person who worked equally on multiple interventions who said, 'retraining or reprogramming program staff to look at recruitment in a different way, not just going to clubs.' And another staff person who was also implementing multiple interventions who said, 'have a continuous ongoing event that we keep our clients connected to the agency. And it's served in different levels: one for the new, to recruit new clients because we ask the clients to bring their friends and their special neighbors; and two for the clients that already went through the intervention.' CDC guidance recommends such quality assurance activities as providing additional staff training and planning for client recruitment and engagement in interventions. A staff person who was implementing Safety Counts noted the sustainability potential of their intervention, 'the other reasons why we continue to use Safety Counts, not only because we also can bring the clients, refer the clients to our organization so it's auto-feeding the medical services that we provide, but also the partnership that we have with other agencies as well'. Segments that were coded as reinvention were not analyzed in this phase because of the small number of coded segments (n = 5).

## Discussion

Modifications to key characteristics, which are considered adaptations necessary to enhance the relevance of the intervention for new settings or populations, were described in each phase of intervention implementation. Reinvention of interventions, which are significant changes to the core elements of the intervention, were also reported within each phase. Staff who implemented interventions were familiar with the core elements of the interventions and recognized when changes to their programs contradicted the internal logic of the intervention. Few participants described piloting of changes before commencing with full implementation of their adaptations as was recommended by CDC. None of the participants reported accessing technical assistance or guidance in making significant changes to their interventions. Despite limited awareness of or access to adaptation technical assistance and guidance, the staff we interviewed executed many of the recommended practices for adapting these interventions for new target populations and new settings. There are however important gaps to be bridged in the technology transfer of these interventions into real-world settings.

### The need for continuous or repeated fidelity monitoring

Continuous or repeated measurements of fidelity to document ongoing modifications to interventions are recommended in light of the ongoing nature of modifications to these interventions. The modification and reinvention examples we have compiled can facilitate program monitoring and fidelity assessments for other CDC-recommended interventions [21,28]. Such monitoring can be incorporated into monthly or periodic reports provided by agencies to their funders to establish a record of the changes made over time and the reasons for such changes. Linking changes within each phase of technology transfer to HIV outcomes among clients served will also permit outcome monitoring of the effectiveness of these programs. In 2010, the CDC will support outcome monitoring and evaluation activities of selected funded HIV prevention programs including selected adapted evidence-based interventions [29].

### The need for strategic technical assistance and guidance on modifications and reinvention

Technical assistance during the pre-implementation phase must shape formative evaluation and piloting among agencies that are modifying key characteristics, such as intervention activities, exercises, and session duration. Focused and explicit technical assistance should be directed to funders who may request content and procedural changes to interventions that contradict core elements and behavioural theory. Future written guidance can include examples of potential activities, content, and exercises that would likely contradict core elements of the recommended interventions. The CDC guidance for the SISTA and Safety Counts interventions already offer clear statements regarding what additions or changes are considered inappropriate [8]. During the implementation phase, technical assistance for agencies can assist staff in developing novel retention strategies including use of social rewards and incentives over monetary incentives, as well as innovative use of the internet for ongoing contacts with intervention participants. Written guidance can identify those situations in which further modifications to an intervention might be warranted, including process monitoring that indicates poor retention or low responsiveness of participants to the intervention. Decisions to make significant changes in this phase should be made in consultation with funders and technical assistance providers. Agencies and funders must be encouraged to evaluate reinvented interventions to demonstrate their ability to reduce risk and address risk factors for HIV. During the maintenance and evolution phase, technical assistance can aid in planning for enhancement of staff skills and expanding client engagement efforts. Modifications and reinvention during this phase must be discussed among agencies, funders, and technical assistance providers if evidence emerges regarding new HIV risk behaviours among target participants and new behavioural risk factors that were not addressed by the original intervention that was selected. Written guidance can encourage agencies to examine possible associations between changes they made to the intervention and increases or decreases in the effectiveness of the intervention as delivered. Such efforts would be consistent with building an evidence base for reinvented interventions within the CDC's Tiers of Evidence framework for classifying interventions [30].

This study contributes to the literature on adaptation of evidence-based intervention through the examination of changes made to multiple HIV prevention interventions for diverse target populations. This complements published work on the diffusion of single interventions across several settings [11,31]. Several limitations of the study deserve note. First, the structured interview questions did not address such important implementation issues as specific program adaptations or the decision-making process by which program administrators selected these adaptations. Greater detail about which activities or sessions were deleted or significantly changed, and the reasons for these changes, may have yielded information regarding low, moderate, or high fidelity of implementation. Second, the absence of actual program monitoring or fidelity assessments of the programs as they were delivered makes it difficult to establish the reliability of the self reports. Participants may have underreported the extent of changes made to their programs because of the emphasis on fidelity to these interventions and concerns about funding. Finally, we did not conduct interviews with staff at different levels of the organization who could have provided corroborating or contrasting information. We plan to conduct research in the future addressing the correspondence between reported adaptations and program fidelity.

## Conclusion

As new evidence-based HIV prevention interventions emerge and are diffused nationally, the number of adapted and reinvented interventions will also grow. Future research can more carefully examine why modifications and reinventions occur and whether such changes were associated with enhanced effectiveness with new target populations or in new settings. Ensuring that fidelity monitoring tools and adaptation technical assistance are made available to implementing agencies and staff when they most need these resources will ensure that HIV prevention technology transfer is considered and measured.

## Competing interests

RCV is a volunteer with the American Psychological Association Behavioral and Social Science Volunteer Program, which is funded by CDC.

## Authors' contributions

RCV supervised all aspects of the interview study and served as the lead writer. UHK conducted interviews, coding of transcripts, and assisted with writing. RR advised on concept, research design, and manuscript editing. All authors have read and approved the final manuscript.
